# Anti-malarial activity and HS-SPME-GC-MS chemical profiling of *Plinia cerrocampanensis* leaf essential oil

**DOI:** 10.1186/1475-2875-13-18

**Published:** 2014-01-13

**Authors:** Armando A Durant, Candelario Rodríguez, Liuris Herrera, Alejandro Almanza, Ana I Santana, Carmenza Spadadora, Mahabir P Gupta

**Affiliations:** 1 Center for Drug Discovery and Biodiversity, Institute of Scientific Research and Technology Services (INDICASAT), Panama, Panama; 2 Center for Cellular and Molecular Biology of Diseases, Institute of Scientific Research and Technology Services (INDICASAT), Panama, Panama; 3 Faculty of Natural, Exact Sciences and Technology, University of Panama, Panama, Panama; 4 Center of Pharmacognostic Research on Panamanian Flora (CIFLORPAN), College of Pharmacy, University of Panama, P.O. Box 0824-00172, Panama, Panama

**Keywords:** Malaria, *Plinia cerrocampanensis*, *Plasmodium falciparum*, Essential oil, Anti-malarial activity, Solid phase microextraction, Gas chromatography-mass spectrometry

## Abstract

**Background:**

*Plinia cerrocampanensis* is an endemic plant of Panama. The leaf essential oil of this plant has shown antibacterial activity. However, anti-malarial activity and chemical profiling by HS-SPME-GC-MS of this essential oil have not been reported before.

**Methods:**

Anti-malarial activity of the essential oil (EO) was evaluated *in vitro* against chloroquine-sensitive HB3 and chloroquine-resistant W2 strains of *Plasmodium falciparum*. Synergistic effect of chloroquine and the EO on parasite growth was evaluated by calculating the combination index. A methodology involving headspace solid phase microextraction and gas chromatography-mass spectrometry (HS-SPME-GC-MS) was developed to investigate the composition of *Plinia cerrocampanensis* EO*.*

**Results:**

*Plinia cerrocampanensis* EO showed a high anti-malarial activity and a synergistic interaction with chloroquine. The *Plinia cerrocampanensis* EO inhibited *P. falciparum* growth *in vitro* at an IC_50_ of 7.3 μg/mL. Chloroquine together with the EO decreased the IC_50_ of chloroquine from 0.1 μg/mL to 0.05 μg/mL, and of the EO from 7.3 μg/mL to 1.1 μg/mL. The measured combination index was 0.58, which clearly indicates that the EO acts synergistically with chloroquine. Since the EO maintained its inhibitory activity on the chloroquine-sensitive strain of the parasite, it could be acting by a different mechanism of action than chloroquine. The best HS-SPME-GC-MS analytical conditions were obtained when the temperature of extraction was 49°C, incubation time 14 min, and the time of extraction 10 min. This method allowed for the identification of 53 volatile constituents in the EO, including new compounds not reported earlier.

**Conclusions:**

The anti-malarial activity exhibited by the *Plinia cerrocampanensis* EO may lend support for its possible use as an alternative for anti-malarial therapy.

## Background

Malaria is a life-threatening tropical disease caused by five species of *Plasmodium*, the most deadly of them in humans being *Plasmodium falciparum*. According to the World Health Organization (WHO), malaria affected 219 million people around the world in 2010, causing over 600,000 deaths. On the other hand, increased rate of resistance to drugs used for treating patients has been observed over the past 20 years [1]. Chloroquine resistance has been reported in most of the malaria-endemic regions, while resistance to artemisinin has been detected in Cambodia, Thailand, Myanmar, and Vietnam [1]. These facts highlight the challenge in discovery of new drugs and new strategies for combating the disease. Historically natural products, such as aromatic plants, have been a source of new drugs and pharmacological alternatives for treating several diseases. The essential oils (EOs) are complex mixtures of terpenes and to a lesser extent of non-terpenoid compounds. Extensive research has been done on chemical characterization and determination of the antibacterial, antifungal, antileishmanial, and antiplasmodial activities of the EOs extracted from a wide variety of plants [2-7].

*Plinia cerrocampanensis* (Myrtaceae) is a tree that grows to a height of c.8 m at an altitude of 800 and 1,000 m in the surroundings of Cerro Campana National Park (Panama) [8]. The EO of this plant has been reported to possess antimicrobial activity against *Staphylococcus aureus, Pseudomonas aeruginosa, Microsporum gypseum, Trichophyton mentagrophytes, Trichophyton rubrum*, and *Helicobacter pylori*[9]. However, its anti-malarial activity has not been reported before. Some terpenes have shown chemotherapeutic activity. For the extraction of the different terpenoids, and other compounds that constitute an EO, head-space solid phase microextraction (HS-SPME) is a powerful tool that requires a minimal amount of sample. The isolated compounds are separated and identified by using GC-MS. SPME, which is a simple, fast, sensitive, solvent-free method developed by Pawliszyn *et al.*[10,11] in the early 1990s, and used for research in many fields, such as cancer [12], medicinal plants [13] and metabolomic analysis [14]. This methodology was used by Da Porto and Decorti [15] for the determination of the aroma profile of the EO obtained by hydro-distillation of lavender (*Lavandula angustifolia*) flowers.

The present study aimed to (a) evaluate *in vitro* anti-malarial activity of *Plinia cerrocampanensis* EO against *P. falciparum*; (b) study the possible synergistic inhibitory effect of chloroquine and the EO on the growth of the parasite; and (c) optimize a new HS-SPME-GC-MS methodology for identification of the volatile constituents of *Plinia cerrocampanensis* EO, which could possibly explain the observed anti-malarial activity.

## Methods

### Plant material and extraction of the essential oil

Fresh leaves of *Plinia cerrocampanensis* were collected in the Altos de Campana National Park (Province of Panama, Panama). The plant was identified by Alex Espinosa, taxonomist of CIFLORPAN, University of Panama. Leaves were protected from humidity and light until extraction of the EO. A voucher specimen No. 6623 is deposited in the Herbarium of the University of Panama (PMA). *Plinia cerrocampanensis* EO was extracted from 100 g of the collected plant material by hydrodistillation, for three hours, using a Clevenger-type apparatus as described in the European Pharmacopeia [16].

### Cytotoxicity assay

Vero cells were seeded in 96-well plates using RPMI 1640 medium (Sigma-Aldrich, USA) supplemented with 10% foetal bovine serum (Gibco, Invitrogen, USA) and a combination of 1% penicillin/streptomycin. The cells were allowed to grow for 24 hours before adding the EO, dissolved in dimethyl sulphoxide (DMSO), to final concentrations of 10, 4, 0.2 and 0.08 μg/mL (w/v). A negative control (with an equal volume of DMSO) was placed in all plates and counted as 100% growth. All samples were incubated for five days before staining and examining for reduction of 3-(4,5-dimethylthiazol-2-yl)-2,5-diphenyltetrazolium bromide (MTT) (Sigma Aldrich, USA) and analysed four hours later in an ELISA plate reader at a wavelength of 570 nm [17].

### Anti-malarial activity

The W2 and the HB3 strains of *P. falciparum* were obtained from the Malaria Research and Reference Reagent Resource Centre (MR4, Manassas, VA, USA), and maintained in continuous culture for about two months before new stocks were thawed. The parasites were grown and maintained in cultures using the method of Haynes *et al.*[18]. All chemicals except sodium bicarbonate and gentamicin (Gibco; Invitrogen, USA), were purchased from Sigma-Aldrich (USA). Cultures consisted of a 2% haematocrit suspension of O^+^ human erythrocytes in RPMI 1640 medium supplemented with a gentamicin solution at 0.01 mg/mL, 25 mM HEPES buffer, 25 mM NaHCO_3_, and 10% human serum. Cultures were fed with a gas mixture consisting of 5% CO_2_, 5% O_2_, 90% N_2_ and incubated in a cyclic incubator to achieve synchronization by temperatures, as described by Almanza *et al.*[19]. Estimation of the parasitaemia as well as visualization of parasite prior to the assays were done using normal light (Giemsa stain) microscopes. All samples were run in duplicate. The assay was carried out in 96-well plates at final concentrations of 10, 2, 0.4 and 0.08 μg/mL and re-evaluated at higher or lower concentrations as necessary. The final dilution contained less than 1% DMSO, which had no measurable effect on parasite survival. DMSO at a final concentration of 1% in RPMI 1640 culture media was used as a negative control. The positive control consisted of chloroquine (CQ) at concentrations of 1,000, 200, 100, 50, and 10 nM. The parasites were incubated for 48 hours at which point 50 μL of a PicoGreen cocktail (Invitrogen, USA) were added to each well and the fluorescence determined after 30 min at 485 nm in a fluorometer. To measure the effect of the samples alone on the fluorescence signal, each extract concentration was incubated in the absence of parasites and the signal was subtracted from the value obtained in the presence of samples and parasites. For the synergistic studies, different doses of CQ (10, 50, 100, 200 and 1,000 nM) and *Plinia cerrocampanensis* EO (0.08, 0.4, 2 and10 μg/ml) were used, maintaining constant the concentration of one while varying the other.

Statistical analysis was carried out with the LSW DATb add-on of Microsoft Office Excel. The significance level was p <0.05.

### Solid phase microextraction fibre selection

Three fibres were evaluated in this research: carboxen-polydimethylsiloxane StableFlex (CAR/PDMS, 75 μm), polydimethylsiloxane-divinylbenzene StableFlex (PDMS/DVB, 65 μm), and divinylbenzene-carboxen-polydimethylsiloxane StableFlex (DVB/CAR/PDMS 50/30 μm) (Supelco, Bellefonte, PA, USA). All fibres were conditioned in the gas chromatograph injector port at the temperature and time recommended by the manufacturer.

Five hundred milligrams of *Plinia cerrocampanensis* EO (liquid matrix) were transferred to a 10 mL vial and hermetically sealed. The sample was equilibrated during 20 min at 30°C in a thermostatic bath. Then the fibre was exposed to sample headspace for 15 min. After the sampling period, the SPME fibre was inserted into the GC injector port and maintained there for 7 min at 250°C for desorption of compounds.

### Experimental design

The times of incubation and extraction and temperature of extraction are critical parameters that determine the equilibrium, vapour pressures and extraction efficiency of volatiles in the headspace [20]. These variables, which strongly influence the efficiency of the HS-SPME-GC-MS, were optimized by using a Doehlert design. Sixteen randomized experiments were performed in duplicate. The total GC-MS peak areas were used as response. Statistical analysis was done using Statgraphics Plus 5.1 (Statistical Graphics, Rockville, MD, USA).

### Gas chromatography-mass spectrometry analysis

All the analyses were performed on an Agilent 6890 N gas chromatograph (GC) equipped with a 5975C mass-selective detector (MSD) with triple-axis detector (Agilent Technologies, Palo Alto, CA, USA). The separation was achieved on a DB5-MS capillary column (30 m length, 0.25 mm id, 0.25 μm film thickness), Agilent Technologies, Palo Alto, CA, USA).

Gas chromatograph oven initial temperature was kept at 50°C for 2 min, then increased to 240°C at 6°C min^-1^ and held for 5 min. The injector temperature was maintained at 250°C, using splitless injection mode (2 min). Helium was used as carrier gas with a constant flow-rate of 1 mL min^-1^. The mass spectrometer was operated in scan mode from 30-550 m/z; ion source temperature was set at 250°C; the ionization was performed in the impact ionization mode (EI) with the ionization voltage set to 70 eV.

Identification of the compounds was carried out based on comparison of their fragmentation patterns using authentic standards when available and the NIST 11 Mass Spectral Search Program (NIST, Washington, USA), and with data from the literature [21]. Further identification of the compounds was achieved by comparison of their calculated experimental linear Kovat’s retention indices (KI) with those reported in the literature using chromatographic columns of similar stationary phase composition.

## Results

### Headspace solid phase microextraction fibre selection

The first HS-SPME parameter optimized for characterization of the metabolites of *Plinia cerrocampanensis* EO was the selection of the SPME fibre. In this study three commercially available SPME fibres, with two or three different coating polymers, were compared for assessing their concentration capacity. SPME fibres with more than one coating have shown to be the most effective when studying medicinal and aromatic plants [22].

The extraction efficiency of each fibre was determined on the basis of two main criteria, the total MS-detector peak area and the number of identified compounds. The DVB/CAR/PDMS fiber permitted the identification of a larger number of constituents of the EO than CAR/PDMS and DVB/PDMS fibers. Furthermore, DVB/CAR/PDMS total peak response (9.12 × 10^10^) was higher than that obtained with DVB/PDMS (8.42 × 10^10^) and CAR/PDMS (4.55 × 10^10^) fibres (Table 1). DVB/CAR/PDMS is an adsorption-based fibre made of polydimethylsiloxane (PDMS) coating that allows the absorption of less polar substances; carboxen (CAR) and divinylbenzene (DVB) porous coating materials that provide high selectivity and sensitivity for more polar compounds. This fibre was selected as the most appropriate for obtaining the widest composition profile of *Plinia cerrocampanensis* EO, and was used in all further experiments.

**Table 1:** **MS detector peak area of some volatile metabolites in *Plinia cerrocampanensis *** **essential oil analysed by using three different SPME fibres**

		**Mean MS response value (×10**^**7**^**)**		
**Compound**		**DVB/CAR/PDMS**	**CAR/PDMS**	**DVB/PDMS**
1	Furfural	67.2	**-**	**-**
2	α-Pinene	584.0	632.0	894.0
3	Benzaldehyde	257.0	**-**	201.0
4	γ-Terpinene	1150.0	799.5	**-**
5	Terpinolene	547.0	**-**	**-**
6	Linalool	2390.0	**-**	2630.0
7	Neo-allo-ocimene	106.0	239.7	30.8
8	4-Terpineol	599.0	467.0	660.0
9	α-Terpineol	231.0	194.0	261.0
10	Nerol	12.9	8.8	15.5
11	Geraniol	26.8	14.3	30.8
12	α-Copaene	663.0	639.0	741.0
13	Sativene	11.1	**-**	**-**
14	α-Cedrene	140.0	**-**	229.0
15	Caryophyllene	114.0	112.0	132.0
16	α-Bergamotene	112.0	105.1	129.0
17	Epi-β-santalene	33.0	**-**	**-**
18	α-Humelene	46.1	46.7	55.5
19	β-Acoradiene	69.2	81.3	93.2
20	γ -Muurolene	89.6	87.2	103.0
21	α-Muurolene	80.3	53.5	96.4
22	α-Curcumene	191.0	170.9	184.0
23	β -Bisabolene	907.0	480.3	1100.0
24	δ-Cadinene	291.0	277.0	328.0
25	*trans*-Nerolidol	195.0	83.8	243.0
26	Cubenol	11.8	8.4	**-**
27	Bisabolol oxide II	37.9	**-**	47.7
28	α-Bisabolol	159.0	58.0	213.0

### Optimization of the HS-SPME extraction procedure

The times of extraction and incubation and temperature of extraction are important factors that determine the efficiency of the overall HS-SPME extraction process [23]. Thus, these parameters were optimized by using a three-variable Doehlert design. This multivariate method allows for the simultaneous evaluation of the factors under study. Table 2 shows the experimental Doehlert design matrix and the response in terms of total area of the compounds identified from *Plinia cerrocampanensis* EO (liquid matrix). The design consisted of 16 randomized experiments to avoid systematic errors. Analysis of variance (ANOVA) was performed for determining the statistical significance (p <0.05).

**Table 2:** **Experimental Doehlert design matrix used for optimization of extraction parameters of *Plinia cerrocampanensis *** **essential oil by HS-SPME**

**Experiment**	**Extraction temperature (°C)**	**Time of equilibrium (min)**	**Time of extraction (min)**	**Total peak area (10**^**9**^**)**
				
1	45	25	20	136.86
2	60	25	20	1.41
3	52	25	35	6.84
4	52	40	25	9.47
5	30	25	20	50.99
6	38	25	5	43.98
7	38	10	15	111.39
8	52	25	5	139.92
9	52	10	15	137.97
10	38	25	35	106.08
11	45	10	30	100.77
12	38	40	25	100.85
13	45	40	10	122.17
14	45	40	10	121.96
15	45	40	10	122.20
16	45	40	10	120.34

**Figure 1:**
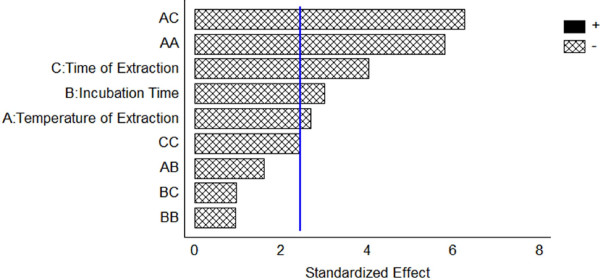
Pareto chart of standardized effects of extraction temperature, time of incubation and time of extraction.

Figure 1 shows the Pareto chart of the standardized effects. As can be observed, all three of the evaluated factors were significant, and increasing their value affected negatively the extraction. Among the HS-SPME parameters studied the time of extraction had the greatest influence on the extraction efficiency, while the temperature of extraction was less significant.

**Figure 2:**
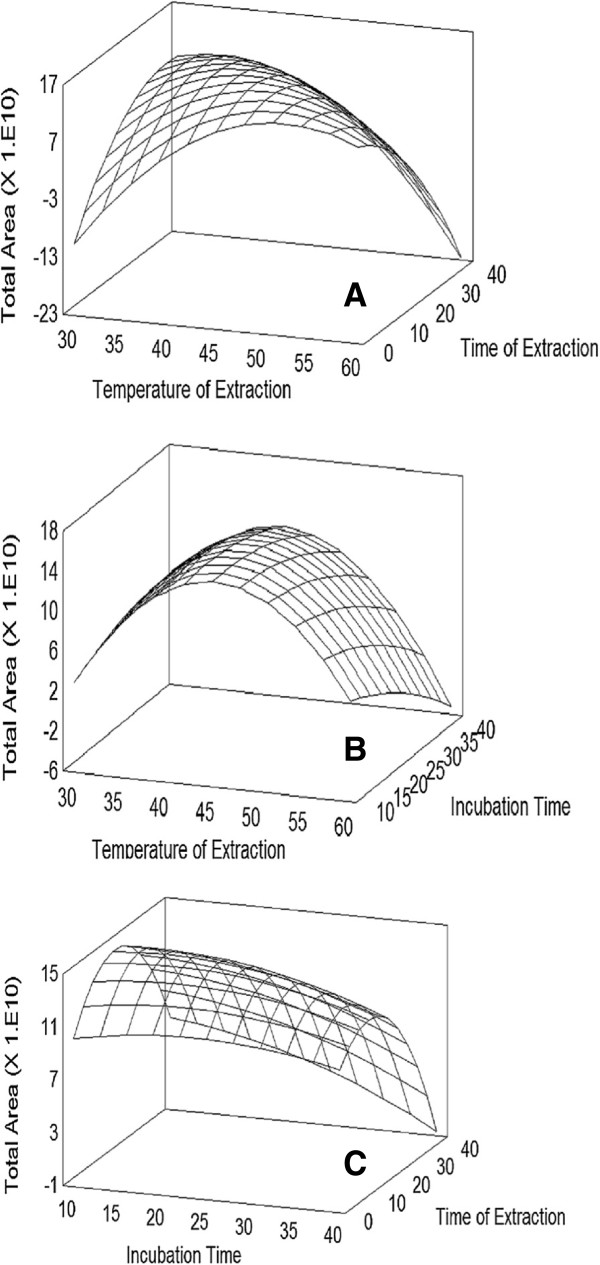
**Fitted response surface plot. (A)** Extraction temperature (°C) *vs* time of extraction (min). **(B)** Extraction temperature (°C) *vs* time of incubation (min). **(C)** Time of incubation (min) *vs* time of extraction (min).

HS-SPME is an equilibrium process among three phases, the sample matrix, a gaseous headspace and the fibre coating. A first equilibrium is established between the analytes in the matrix and the headspace; subsequently, the SPME fibre is introduced in the headspace leading to a new equilibrium between the analytes in the headspace and in the fibre [24]. By raising the temperature the amount of analytes released from *Plinia cerrocampanensis* EO and extracted by the SPME fibre increased until an optimal value at 49°C (Figure 2A), after which higher temperatures influenced negatively the results. As the extraction temperature increases the Henry’s constant of the analytes also increases, as well as the amount of compounds released from the liquid phase, especially less volatile compounds. Both physicochemical phenomena improve the extraction efficiency. Nevertheless, above 49°C the amount of compounds extracted by the SPME_DVB/CAR/PDMS_ fibre tends to decrease due to desorption of compounds from the fibre.

Incubation time influences the number of compounds that are transferred from the liquid matrix and reach the headspace. The effect of this variable on analytical response is shown in Figure 2. A maximum response was observed at 14 min. No improvement on extraction of EO metabolites was observed by increasing the incubation time.

In order to assess the effect of extraction time on the analysis, the SPME_DVB/CAR/PDMS_ fibre was introduced into the sample headspace for different periods. The optimal response was achieved with an extraction time of 10 min (Figure 2). HS-SPME is an equilibrium process influenced strongly by the partition coefficient of the compounds between the liquid phase and the headspace (K_fiber/matrix_).

Withdrawing the SPME fibre from the headspace before 10 min did not allow this equilibrium to be achieved, and hence less metabolites were extracted. Conversely, higher time of extraction reduced the total volatile recovery due to diffusion of the analytes from the fibre [25].

According to the experimental results, the best analytical response was obtained when the temperature of extraction was 49°C, incubation time 14 min, and time of extraction 10 min. These optimal values were selected for extraction of the EO metabolites in all further experiments.

### Chemical composition of the essential oil of *Plinia cerrocampanensis* by HS-SPME-GC-MS

This is the first time that the chemical composition profiling of *Plinia cerrocampanensis* EO has been studied by using HS-SPME-GC-MS approach. Table 3 lists the constituents identified in the EO analysed. Fifty-three compounds were identified by this methodology. The main constituents (more than 2% of the identified compounds) were β-bisabolene, β-linalool, α-copaene, *o-*cymene, γ- terpinene, δ-cadinene, α-curcumene, α-bisabolol, neo-allo-ocimene, *trans*-nerolidol, *trans*-β-ocimene, β-ocimene, γ-bisabolene and terpinolene (Table 3). Vila *et al.* have reported the chemical composition of the EO obtained from leaves of *Plinia cerrocampanensis* utilizing two different GC-columns, i e, a non-polar and a polar phase columns, identifying 40 compounds: 33 compounds were identified on the non-polar phase column, and 31 on the polar phase column [9]. Clearly, the more sensitive HS-SPME-GC-MS methodology developed in this study allowed the identification of a higher number of compounds in a single chromatographic run.

**Table 3:** **Chemical composition of *Plinia cerrocampanensis *** **B. essential oil by HS-SPME-GC-MS**

**Peak no**	**Compound**	**KI**^**a**^	**m/z**	**Percentage composition (%)**
1	Furfural	830	95-67–42	0.21
2	α-Pinene	933	93–79–41	1.96
3	Benzaldehyde	958	105–77–51	1.10
4	Myrcene	982	41–93–69	0.91
5	3-δ-Carene	1006	93–79–41	0.90
6	*o*-Cymene	1020	119–91–134	6.85
7	*trans*-β-Ocimene	1041	93–79–41	2.62
8	*cis*-β-Ocimene	1053	93–91–79	2.58
9	γ-Terpinene	1067	93–77–41	4.70
10	Terpinolene	1092	93–121–136	2.01
11	Linalool	1104	43–71–55	10.47
12	Fenchol	1121	81–43–67	0.06
13	*cis*-Sabinol	1133	92–81–109	0.11
14	Neo-allo-ocimene	1140	121–105–79	2.72
15	1,4-Dimethyl-4-acetyl-1-cyclohexene	1157	109–67–43	0.04
16	*Trans,*cis-2,6-Nonadienal	1163	41–70–39	0.05
17	Terpin-4-en-1-ol	1174	43–71–93	2.22
18	*m*-Methylacetophenone	1188	119–91–65	0.15
19	α-Terpinol	1196	59–43–93	1.20
20	β-Cyclocitral	1214	41–137–123	0.06
21	Nerol	1229	41–69–81	0.12
22	*p*-Menth-1(7)-en-2-one	1241	82–81–152	0.07
23	Carvenone	1248	95–110–67	0.18
24	Geraniol	1255	41–69–81	0.29
25	*cis*-2-Decenal	1272	41–55–70	0.04
26	Carvacrol	1313	135–91–150	0.05
27	α-Copaene	1365	105–119–161	8.86
28	Sativene	1390	108–41–91	0.19
29	Ylangene	1400	105–119–93	0.89
30	α-Cedrene	1412	119–41–105	0.92
31	*trans*-β-Caryophyllene	1420	41–91–79	1.34
32	α-Bergamotene	1440	41–93–119	1.93
33	γ-Elemene	1449	121–41–93	1.24
34	Epi-β-santalene	1453	94–41–79	0.52
35	α-Humelene	1458	93–41–67	0.54
36	β-Farnesene	1462	41–69–91	1.97
37	β-Acoradiene	1469	119–41–105	1.05
38	γ-Gurjunene	1476	105–91–81	0.82
39	γ -Muurolene	1484	161–41–105	1.42
40	α-Curcumene	1490	119–132–41	3.83
41	Indipone	1499	149–93–41	1.18
42	α-Muurolene	1507	105–41–161	0.95
43	β-Bisabolene	1513	41–69–93	16.26
44	δ-Cadinene	1527	119–161–41	4.21
45	γ-Bisabolene	1540	41–93–107	2.19
46	trans- Nerolidol	1558	41–69–93	2.68
47	Spathulenol	1569	43–91–79	0.27
48	tau-Cadinol	1632	43–161–81	0.26
49	Cubenol	1649	43–161–95	0.26
50	Bisabolol oxide B	1666	43–59–105	0.92
51	β-Bisabolol	1674	41–67–93	0.15
52	α-Bisabolol	1687	43–119–109	3.46
53	α-Farnesol	1709	41–69–81	0.01

^a^Retention index. Components are listed in increasing order of their retention indices on DB5-MS.

The current investigation reports for the first time new compounds present in the EO obtained from leaves of *Plinia cerrocampanensis*, such as neo-allo-ocimene, γ-elemene, caryophyllene, acoradiene, γ-muurolene, and β-myrcene.

Sesquiterpene hydrocarbons (49.13%), and monoterpene hydrocarbons (25.25%) were the most abundant chemical class of compounds identified by HS-SPME-GC-MS, followed by oxygenated monoterpenes (15.03%), oxygenated sesquiterpenes (9.19%) and aldehydes (1.35%).

### Anti-malarial activity

This is the first report on the anti-malarial activity of the EO from *Plinia cerrocampanensis*. The antiplasmodial effects of *Plinia cerrocampanensis* EO were determined by quantifying the growth inhibition of the CQ-resistant *P. falciparum* W2 strain when compared with parasites cultured in a medium free of the EO. The *Plinia cerrocampanensis* EO inhibited parasite growth *in vitro* at IC_50_ 7.3 μg/mL. This value indicates a high antiplasmodial activity. The activity of the oil was lower than that of chloroquine (IC_50_ = 0.1 μg/mL), which could result from several factors, one of them being the dilution of the active compound (s) in the matrix.

To get an idea of the mechanism by which the EO was acting on *P. falciparum*, its activity against a CQ-sensitive strain, HB3, was tested and compared the results with those obtained with the CQ-resistant W2. While CQ shows difference in IC_50_ in the resistant (IC_50_ = 0.1 μg/mL) and sensitive (IC_50_ = 0.01 μg/mL) strains, the EO maintained its level of activity, in both the resistant (IC_50_ = 7.3 μg/mL) and sensitive (IC_50_ = 10.2 μg/mL) strains. These results suggest that the EO may be using another mechanism that does not involve inhibition of β-haematin formation nor inhibition of the peroxidative degradation of haemin, since CQ and other quinolones with anti-malarial activity are thought to act through these two mechanisms [26,27].

The EO caused toxicity to mammalian cells at a concentration of 28.6 μg/mL, which is four-fold higher than the concentration at which the oil showed antiplasmodial effects, projecting a reasonable therapeutic window for its use *in vivo*. The HS-SPME-GC-MS methodology developed in this study allowed the identification of terpenes and terpenoids in the EO of *Plinia cerrocampanensis*: α-pinene, β-linalool, *trans*-nerolidol, α-bisabolol and α-farnesol, which have been reported to possess antiplasmodial activity and, therefore, may explain the observed anti-malarial activity of EO [28-30].

The monoterpene α-pinene displayed a stronger anti-malarial activity than quinine when tested against a CQ-resistant strain of *P. falciparum*, mainly affecting the ring stage of the parasite [29]. β-linalool and farnesol arrest the growth of the apicomplexan mainly by (a) affecting the dolichol biosynthesis at the trophozoite and schizont stages; and, (b) inhibiting the biosynthesis of the isoprene side chain of the benzoquinone ring of ubiquinones [30]. Treatment of *P. falciparum* with the isolated oxygenated sesquiterpene α-bisabolol caused inhibition in the development of parasites [30]. Macedo *et al.* demonstrated the high activity of the sesquiterpene *trans*-nerolidol against *P. falciparum* by decreasing its ability to biosynthesize the isoprene chain attached to coenzyme Q [31]. In most cases, the anti-malarial activity displayed by the *Plinia cerrocampanensis* EO was higher than the activity observed for these compounds, suggesting that trace components of the oil could be enhancing the effect of the active components. A possible synergism among the different constituents of the oil could also be responsible for its anti-malarial activity.

**Figure 3:**
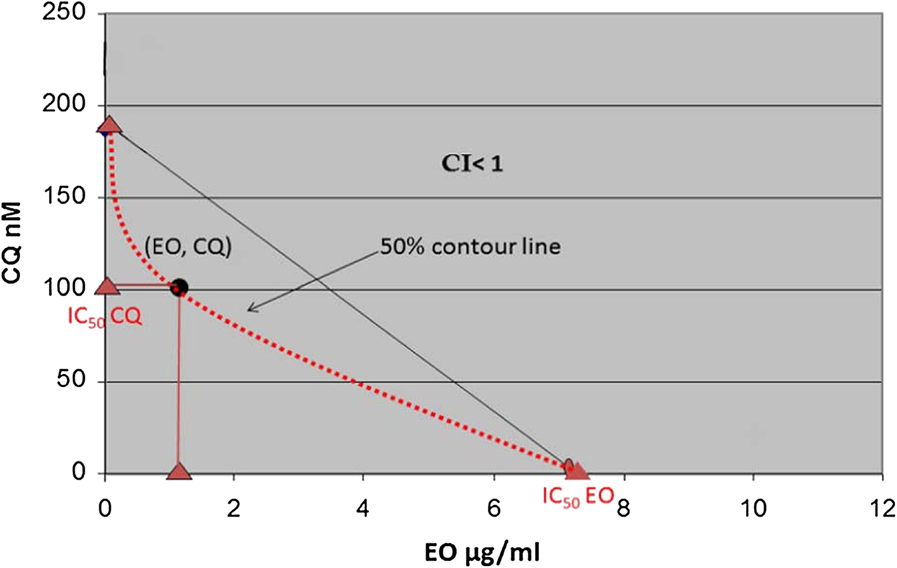
**Isobologram and combination index (CI) of chloroquine (CQ) and the essential oil (EO) of*Plinia cerrocampanensis*.** The 50% contour line shows a less than 1 combination index which shows synergy between EO and CQ at concentration (EO, CQ).

To determine possible synergistic or additive effects when applied with other drugs, the activity of the EO was examined in conjunction with CQ at different concentrations. Synergism or additive effect was evaluated by calculating the combination index (CI) as described by Chou and Talahay [32] using the following formula:

$$\mathrm{CI}=\frac{{\left(D\right)}_{1}}{{\left(D_{x}\right)}_{1}}+\frac{{\left(D\right)}_{2}}{{\left(D_{x}\right)}_{2}}=\frac{1}{{\left(\mathrm{DRI}\right)}_{1}}+\frac{1}{{\left(\mathrm{DRI}\right)}_{2}}$$

where D is the concentration at which both compounds show the same desired efficacy (ED) in combination, and D_x_ is the concentration at which each of the compounds alone show this ED [32]. A synergistic effect is achieved if CI is <1.0 while an additive effect is obtained at CI = 1.0. The application of CQ together with the EO on strains of *P. falciparum*, decreased the IC_50_ for CQ from 0.1 μg/mL to 0.05 μg/mL, and for the EO from 7.3 μg/mL to 1.1 μg/mL (Figure 3).

This means that a higher *in vitro* activity against the parasite was obtained for the oil and CQ when applied together. The measured CI combination was 0.58, which clearly indicates that the EO acted synergistically with CQ. Moreover, the therapeutic window of the EO increased substantially in the presence of a very small amount of CQ. Based on the physico-chemical properties of the EO, it could also cause the disruption of the parasite cell membrane, which affects the permeability of the cell and the transfer of important biochemical substances needed by the parasite for sustaining crucial metabolic processes. The reduction in the concentration of CQ required for achieving its therapeutical effect may not only lower the incidence of adverse reactions in humans, but most importantly, may promote the use of this cost effective drug in the large regions worldwide where resistance to it has been observed.

## Conclusions

This research demonstrated that the EO of *Plinia cerrocampanensis* could be a potential alternative for the treatment of malaria. Moreover, due to the synergistic anti-malarial activity of the EO and the CQ, this could be used in combination with CQ as anti-malarial therapy. The studies are underway to determine the anti-malarial effect of major isolated compounds in the EO of *Plinia cerrocampanensis*.

The developed HS-SPME-GC-MS methodology allowed for the identification of new constituents in the EO of *Plinia cerrocampanensis*. Monoterpenes and sesquiterpenes, which constitute most of the volatile organic compounds of the essential oil, could be responsible for the bioactivity showed by the oil in this study.

## Abbreviations

EO: Essential oil; HS-SPME: Headspace-solid phase microextraction; GC: Gas chromatography; MS: Mass spectrometry.

## Competing interests

The authors declare that they have no competing interests.

## Authors’ contributions

CS, MPG and AAD participated in research design and critical review of the manuscript; MPG supplied plant material and suggested this research; AIS, CR, LH, AA and AAD conducted experiments; AIS, CS and AAD performed data analysis; CS and MPG contributed in the writing and revision of the manuscript; AAD drafted and wrote the final manuscript. All authors approved the final manuscript.
