# Correction: Anti-malarial activity and HS-SPME-GC-MS chemical profiling of *Plinia cerrocampanensis* leaf essential oil

**DOI:** 10.1186/1475-2875-13-235

**Published:** 2014-06-14

**Authors:** Armando A Durant, Candelario Rodríguez, Liuris Herrera, Alejandro Almanza, Ana I Santana, Carmenza Spadafora, Mahabir P Gupta

**Affiliations:** 1 Center for Drug Discovery and Biodiversity, Institute of Scientific Research and Technology Services (INDICASAT), Panama, Panama; 2 Center for Cellular and Molecular Biology of Diseases, Institute of Scientific Research and Technology Services (INDICASAT), Panama, Panama; 3 Faculty of Natural, Exact Sciences and Technology, University of Panama, Panama, Panama; 4 Center of Pharmacognostic Research on Panamanian Flora (CIFLORPAN), College of Pharmacy, University of Panama, P.O. Box 0824–00172, Panama, Panama

## Correction

The authors would like to publish a correction article to address the misspelling of co-author Carmenza Spadafora’s name in the original article published in *Malaria Journal*[1] and also include further details on funding in the acknowledgements section.
